# Use of Behavioral Change Techniques in Web-Based Self-Management Programs for Type 2 Diabetes Patients: Systematic Review

**DOI:** 10.2196/jmir.2800

**Published:** 2013-12-13

**Authors:** Michael van Vugt, Maartje de Wit, Wilmy HJJ Cleijne, Frank J Snoek

**Affiliations:** ^1^Department of Medical PsychologyVU University Medical CentreAmsterdamNetherlands; ^2^EMGO+ Institute for Health and Care ResearchVU University Medical CentreAmsterdamNetherlands

**Keywords:** Web-based, online, self-management, review, type 2 diabetes mellitus, behavioral change techniques

## Abstract

**Background:**

Type 2 diabetes mellitus (T2DM) is a highly prevalent chronic metabolic disease characterized by hyperglycemia and cardiovascular risks. Without proper treatment, T2DM can lead to long-term complications. Diabetes self-management is recognized as the cornerstone of overall diabetes management. Web-based self-management programs for T2DM patients can help to successfully improve patient health behaviors and health-related outcomes. Theories can help to specify key determinants of the target behaviors and behavior change strategies required to arrive at the desired health outcomes, which can then be translated into specific behavioral techniques or strategies that patients can learn to apply in their daily life. From previous reviews of a wide range of online diabetes self-management tools and programs, it appears that it is still unclear which behavioral change techniques (BCTs) are primarily used and are most effective when it comes to improving diabetes self-management behaviors and related health outcomes.

**Objective:**

We set out to identify which BCTs are being applied in online self-management programs for T2DM and whether there is indication of their effectiveness in relation to predefined health outcomes.

**Methods:**

Articles were systematically searched and screened on the mentioned use of 40 BCTs, which were then linked to reported statistically significant improvements in study outcomes.

**Results:**

We found 13 randomized controlled trials reporting on 8 online self-management interventions for T2DM. The BCTs used were feedback on performance, providing information on consequences of behavior, barrier identification/problem solving, and self-monitoring of behavior. These BCTs were also linked to positive outcomes for health behavior change, psychological well-being, or clinical parameters.

**Conclusions:**

A relatively small number of theory-based online self-management support programs for T2DM have been reported using only a select number of BCTs. The development of future online self-management interventions should be based on the use of theories and BCTs and should be reported accurately.

## Introduction

Type 2 diabetes mellitus (T2DM) is a chronic metabolic disorder characterized by insulin resistance and beta cell impairment [1]. The number of people with T2DM is rising exponentially and is estimated to reach 439 million patients worldwide in 2030 [2]. Without proper treatment, T2DM can lead to long-term complications, such as neuropathy, nephropathy, retinopathy, cardiovascular disease, and a lowered quality of life [3]. The treatment of T2DM patients is largely dependent on the patient’s daily self-care by means of lifestyle modification (diet and physical exercise) and taking oral blood glucose-lowering medication and/or insulin, often combined with medication to normalize blood pressure, cholesterol, and triglycerides [4,5]. Therefore, diabetes self-management is recognized as the cornerstone of overall diabetes management [6,7].

Self-management enables patients to take control of their chronic disease, such as the treatment and the physical and psychological symptoms, by making their own decisions and performing self-chosen actions aimed at improving their health [8-10]. For T2DM, the Association of American Diabetes Educators (AADE) has defined 7 key self-management behaviors: (1) healthy eating, (2) being active, (3) monitoring, (4) taking medication, (5) problem solving, (6) reducing risks, and (7) healthy coping [11].

To promote daily self-management for T2DM patients, educational and behavioral support programs have been developed and shown to be effective for behavioral and medical outcomes [7,12-15]. More recently, self-management programs for T2DM patients are also available on the Internet [16-19]. Web-based self-management programs for T2DM patients have been shown to increase the effectiveness and reach of clinical-based consultations [20]. Furthermore, these Web-based programs can help to improve patient health behaviors (eg, self-monitoring, physical activity, diet) and subsequent health outcomes (eg, weight, glycemic control, emotional distress) [21,22]. However, attrition can be problematic in Web-based interventions and should be considered during the creation process [23].

It is recognized that theory-based self-management programs are more effective than non-theory-based programs; indeed, most self-management programs are informed by theory or elements of a behavior change model [10,24,25]. Theories can help to specify key determinants of the target behaviors and behavior change strategies required to arrive at the desired health outcomes, which can then be translated into specific behavioral techniques or strategies that patients can learn to apply in their daily life [8]. Abraham and Michie [26,27] have developed a taxonomy of behavioral change techniques (BCTs) for different health behaviors, such as healthy eating and physical exercise. Such taxonomy can help to identify successful BCTs and support the development of new online self-management programs for T2DM and other chronic diseases [25-27]. From previous reviews of a wide range of online diabetes self-management tools and programs, it would appear that it is still unclear which BCTs are most used and most effective when it comes to improving self-management behaviors and related health outcomes [21,22,28,29]. Therefore, we set out to: (1) systematically review the literature and identify which BCTs are being applied in online self-management programs for T2DM and how often, and (2) determine whether there is indication from randomized controlled trials (RCTs) for the effectiveness of applied BCTs in relation to particular health outcomes.

## Methods

### Search

On July 24, 2012, we searched within PubMed, EMBASE, Cochrane, PsycInfo, and Cinahl. Because of the size of the search term used, the search terms can be found in Multimedia Appendix 1. Some keywords used in the search were diabetes mellitus; diabetes mellitus, type 2; Internet; eHealth; online; and Web-based. The systematic review was conducted following the Preferred Reporting Items for Systematic Reviews and Meta-Analyses (PRISMA) statement where applicable [30].

### Inclusion Exclusion Criteria

The retrieved articles were screened using the following inclusion criteria: written in English, published after 1994 (the introduction of the Internet), about T2DM, included patients aged 18 years or older, and concerned Web-based (online) self-management programs for which participants had to use the Internet to connect to the intervention. We only included RCTs to establish whether the BCTs used in the programs were associated with significant improvements. We defined self-management programs as systematic approaches to assist patients in their diabetes self-care, and where in some way or other patients were actively engaged and prompted to make decisions for themselves and have responsibility over their own actions [8,10]. Articles were excluded if they were not related to diabetes, reported only on technology testing, were not Web-based programs, did not target a self-management behavior, or only included type 1 diabetes mellitus (T1DM). Book chapters, abstracts, and pilot studies were not included.

### Study Selection and Data Extraction

Two researchers (MvV, WHJJC) independently reviewed the articles and extracted data on demographics, care setting, type of study, duration, measurements, nature of the intervention and control condition, applied inclusion criteria, used theory or model, BCTs, target behavior(s), outcome parameters, results, limits, and adherence. The risk of bias was assessed for all included studies using a quality assessment tool as proposed by van Tulder et al [31] and can be found in the Multimedia Appendix 2. The BCTs were categorized based on the checklist as proposed by Michie et al [27] which can be found in Multimedia Appendix 3. Disagreements regarding defined BCTs between the researchers were resolved by discussion within the research group. The BCTs used and the statistically significant outcomes were uncovered for each study. For each study with an improved study outcome (health behaviors, clinical outcome measures, and psychological outcomes), we looked if a BCT was present in the intervention for improving that particular outcome. We used Microsoft Excel 2003 to cross-reference this data and generated a list of frequently used BCTs associated with significant improvements in defined behavioral, clinical, and psychological outcomes.

## Results

### Article Selection

**Figure 1:**
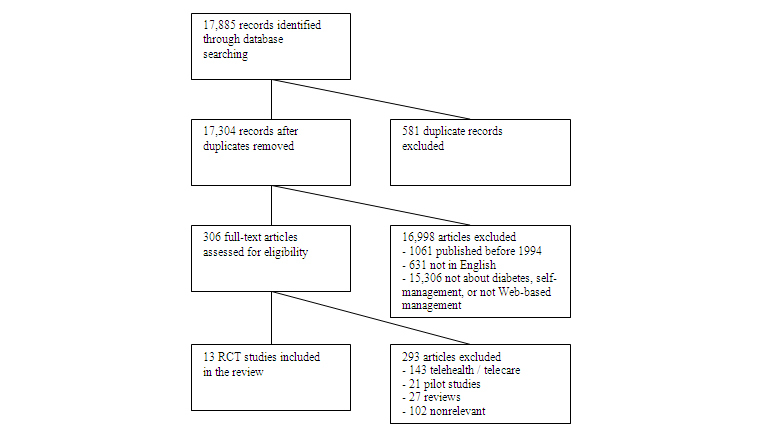
Flowchart screening process of articles included in review.

Figure 1 shows a flowchart of the screening process. The search query resulted in 17,885 articles. After removing duplicated articles, titles and abstracts were screened for inclusion and exclusion criteria. After the first draft, 16,998 articles were excluded because they did not meet the inclusion criteria. We categorized the remaining 306 articles as: (1) studies on Web-based self-management programs, (2) reviews, (3) telehealth, telecare, or telemedicine studies [32-34], and (4) nonrelevant studies. This resulted in 13 articles reporting on 8 different Web-based self-management interventions for T2DM patients. These articles were individually read, screened for the BCTs used, and then discussed to reach consensus. Most articles provided only a short basic description of the intervention that was used. For 1 study [35], an additional article was consulted to uncover the content of the intervention [36].

### Study Characteristics

Tables 1 and 2 provide an overview of the included studies and their results. Of the 13 RCT studies, 10 were performed in North America [37-46], 1 in Asia [47], and 2 in Europe [35,48]. The combined total patient sample size was 3813. Demographically, on average 54.8% of the participants were female and the average age was 57.2 years (SD 7.20). Average program completion rate was 81.7% (SD 15.2%). Four studies recruited their participants from the community by using flyers and newspapers [35,39,45,48]. Five studies recruited their participants from primary health care [37,42-44,46], 1 study recruited their participants from secondary health care [47], and 3 studies recruited their participants from primary and secondary health care [38,40,41]. All the studies included patients who had been diagnosed with T2DM for longer than a year. Five studies also included patients with T1DM [35,38,40,41,48] and 1 study also offered the intervention to people with diagnoses of chronic heart disease and chronic lung disease [45]. Average study duration was 6.69 months (SD 4.92). Adherence for all studies was high, which in itself contributes to the overall high quality of the included studies.

**Table 1:** Characteristics of the studies.

Study	Study description								Participants		
	Quality^a^	Country	n	Groups, n	Measurements, n	Setting^b^	Inclusion criteria^c^	Duration (months)	Ethnicity	Female, %	Age, mean
Glasgow et al (2012) [37]	83%	US	463	3	3	1	T2DM, age 25-75, BMI >25 kg/m^2^, at least 1 other risk factor for heart disease, access to telephone and Internet, fluent in English or Spanish, ability to perform mild to moderate exercise	12	White, Latino	50	58.4
Van Bastelaar et al (2011) [35]	83%	NL	255	2	3	1+2	CESD>16, email address, access to Internet, no history of suicide, suicidal ideation, bipolar, psychotic, pregnancy, recent loss of significant other	3	White	61	50.0
Bond et al (2010) [38]	72%	US	62	2	2	1+2	T1DM or T2DM for at least 1 y, age ≥60, living independently, fluent in English	6	White	45	67.2
Lorig et al (2010) [39]	78%	US	761	3	3	1+2	T2DM, age ≥18, not pregnant or in care for cancer, access to the Internet	18	White, Native Indian, Alaska Native	73	54.3
Glasgow et al (2010) [46]	78%	US	463	3	2	1	T2DM, age 25-75,BMI>25 kg/m^2^, at least 1 other risk factor for heart disease, access to telephone and Internet, fluent in English or Spanish, ability to perform mild to moderate exercise	4	White	50	58.4
Wangberg et al (2008) [48]	67%	NO	61	2	2	1+2	T1DM or T2DM, access to Internet (no exclusion criteria)	1	White	57	40.1
Bond et al (2007) [40]	78%	US	62	2	2	1+2	T1DM or T2DM for ≥1 y, age ≥60, living independently, fluent in English	6	White	45	67.2
Bond et al (2006) [41]	50%	US	15	2	2	1+2	T1DM or T2DM for ≥1 y, age ≥60, living independently, fluent in English	6	White	—	—
Kim et al (2006) [47]	72%	KR	73	3	2	2	T2DM <20 y, age ≥20, FBS <240 mg/dL and/or HbA1c less than 10.0%, no chronic complications, no evidence of heart disease, musculoskeletal disorders, or other disabling diseases that could restrict physical activity, no insulin administration	3	Asian	47	55.1
Lorig et al (2006) [45]	78%	US	958	2	3	1+2	Age ≥18, T2DM or COPD or CHF, no active treatment of cancer for 1 y, not participated in self-management program, access to Internet (email), agree to 1-2 h per week of log-on time over at least 3 sessions/w for 6 w, able to complete the online questionnaire	12	White	71	57.5
Glasgow et al (2003) [42]	67%	US	320	3	2	1	T2DM (Welborn criteria), age 40-75, have a telephone, fluent in English, live in local area and planning to remain in the area for year of study	10	White	53	59.0
Barrera et al (2002) [43]	72%	US	160	4	1	1	T2DM (Welborn criteria), age 40-75, have a telephone, fluent in English, live in local area and planning to remain in the area for year of study	3	White	53	59.0
McKay et al (2002) [44]	72%	US	160	4	2	1	T2DM (Welborn criteria), age: 40-75, have a telephone, fluent in English, live in local area and planning to remain in the area for year of study	3	White	53	59.3

^a^Assessment of study quality as proposed by van Tulder et al [31] see Multimedia Appendix 2.

^b^1=Primary care setting; 2=secondary care setting.

^c^CESD: Center for Epidemiologic Studies Depression Scale; FBS: fasting blood sugar; HbA1c: glycated hemoglobin; COPD: chronic obstructive pulmonary disease; CHF: congestive heart failure; T2DM: type 2 diabetes mellitus; T1DM: type 1 diabetes mellitus.

**Table 2:** Results of the studies.

Study	Results^a^	Completion rate (adherence)	Power calculation
Glasgow et al (2012) [37]	Significant improvements in diet (fat intake), physical activity, and biological outcomes in both IGs vs baseline, and significant reduction in distress for both groups vs CG	77%	Yes
Van Bastelaar et al (2011) [35]	Significant improvements in depression and diabetes distress for IG	68%	Yes
Bond et al (2010) [38]	Significant improvements in quality of life, depression, social support, and self-efficacy for IG	100%	Yes
Lorig et al (2010) [39]	Significant improvements in HbA1c, patient activation, and self-efficacy for IGs vs CG	82%	Yes
Glasgow et al (2010) [46]	Significant improvements in diet (fat intake), physical activity, and biological outcomes in both IGs vs baseline, and significant reduction in distress for both groups vs CG	83%	Yes
Wangberg et al (2008) [48]	Significant improvements in self-care for high-efficacy group	45%	Yes (after)
Bond et al (2007) [40]	Significant improvements in HbA1c, weight, and HDL cholesterol for IG vs CG	100%	Yes
Bond et al (2006) [41]	Significant improvements in HbA1c and high comorbidities for IG vs CG	—	Yes
Kim et al (2006) [47]	Significant improvements in physical activity, FBS and HbA1c for both IGs vs CG	100%	Yes
Lorig et al (2006) [45]	Significant improvements in exercise, health distress, fatigue, pain, shortness of breath, reduction in disability for IG	82%	No
Glasgow et al (2003) [42]	Significant improvements in psychosocial and some biological outcomes for all IGs vs CG	82%	No
Barrera et al (2002) [43]	Significant improvements in diabetes-specific support measure and a general support scale for all IGs vs CG	79%	No
McKay et al (2002) [44]	Significant improvements in diet for all IGs vs CG, but no significant differences between conditions	84%	No

^a^IG: intervention group; CG: control group; HDL: high-density lipoprotein; FBS: fasting blood sugar; HbA1c: glycated hemoglobin.

### The Interventions

Tables 3 and 4 provide an overview of the interventions. Four of the 8 identified online interventions were developed by adapting existing (group) self-management programs into online self-management programs [35,39,45,47], and 4 interventions were newly created [37,38,40-44,46,48]. Two self-management interventions were developed as adjuncts to routine diabetes care, in which health care providers were able to have either online synchronous and asynchronous communication or telephone contact with the patient [38,40,41,47]. Six interventions were developed as standalone programs [35,37,39,42-46,48]. Five interventions were structured as sequential lessons [35,37,39,45-47] and 3 interventions allowed the participant to navigate freely through the program [38,40-44,48]. All 8 interventions offered some form of online coaching [35,37-48]. Seven of the 8 programs reported using a psychological theory or model as the basis for the self-management program, where some programs used multiple theories [37,42,46]. The theories and models used were: self-efficacy theory, [39,42,45], social support theory [42], transtheoretical model (TTM) [47], social cognitive theory [37,46,48], social-ecological model [37,46], and cognitive behavioral therapy [35].

**Table 3:** Characteristics of interventions and control conditions of the intervention programs.

Study	Intervention condition 1	Intervention condition 2	Intervention condition 3	Control condition
Glasgow et al (2012), Glasgow et al (2010) [37,46]	Self-administered, Web-based diabetes self-management program with goal setting and action planning for medication adherence, physical activity, and diet; self-monitoring and feedback on progress, monitoring of blood glucose, blood pressure, and cholesterol results, a moderated forum with community resources, and barrier identification	Self-administered, Web-based diabetes self-management program with goal setting and action planning on medication adherence, physical activity, and diet; self-monitoring and feedback on progress, monitoring of blood glucose, blood pressure, and cholesterol results; a moderated forum with community resources, and barrier identification; 2 follow-up calls from interventionist and invitation to attend 3 group sessions	—	Enhanced usual care (computer-based health risk appraisal feedback and recommended preventive care behaviors)
van Bastelaar et al (2011) [35]	Eight online lessons with cognitive behavioral therapy, coaching feedback, and mood diary	—	—	Waitlist control
Bond et al (2010), Bond et al (2007), Bond et al (2006) [38,40,41]	Online library, a personal electronic log of self-management activities, advice and counseling from a nurse via email, and weekly online problem-solving group discussions	—	—	Usual care
Lorig et al (2010) [39]	Web-based diabetes self-management program, 6 weekly sessions, bulletin board feedback on action planning, problem solving, difficult emotions, and celebrations	Web-based diabetes self-management program, 6 weekly sessions, bulletin board feedback on action planning, problem solving, difficult emotions and celebrations,. listserve discussion group	—	Usual care
Wangberg et al (2008) [48]	Intervention tailored to high self-efficacy aimed at self-care: blood glucose monitoring, diet and physical activity, included T2DM information, barrier identification, quizzes with feedback, videos of peers, video lectures of professionals	Intervention tailored to low self-efficacy, aimed at self-care blood glucose monitoring, diet, and physical activity, including T2DM information, barrier identification, quizzes with feedback, videos of peers, video lectures of professionals	—	Usual care
Kim et al (2006) [47]	Web-based tailored physical activity counseling, based on participants’ assessed motivational stage	Printed-material physical activity intervention including the 5 stages of motivation change	—	Usual care
Lorig et al (2006) [45]	Web-based bulletin board discussion groups and a book, program contains individual exercise programs, emotion management, overview of medications, communication, healthy eating, fatigue management, action planning, feedback, and problem solving	—	—	Usual care
Glasgow et al (2003), Barrera et al (2002), McKay et al (2002) [42-44]	Goal setting with personalized feedback, barrier identification and problem solving, personalized self-management coach condition	Goal setting with personalized feedback, barrier identification and problem solving, peer support condition	Combined condition	Only diabetes information online condition

**Table 4:** Characteristics of the intervention programs.

Study	Theory used	BCTs^a^	Health care professional included	Evolved or new intervention	Standalone or embedded in care
Glasgow et al (2012), Glasgow et al (2010) [37,46]	Social cognitive theory, social-ecological model	1,2,4,5,7-10,13,16,17,19,29,35	No	New	Standalone
van Bastelaar et al (2011) [35]	Cognitive behavioral therapy	1,2,4,8,12,13,15,16,19,21,22,24, 26,27,29,35,36,39,40	No	Evolved	Standalone
Bond et al (2010), Bond et al (2007), Bond et al (2006) [38,40,41]	—	1,2,5,6,8,10,11,16,17,19,21,23, 26,28,30,36	No	New	Embedded in care
Lorig et al (2010) [39]	Self-efficacy theory	1-3,7,8,10,16,17,19,28,29,36	No	Evolved	Standalone
Wangberg et al (2008) [48]	Social cognitive theory	1,2,8,16,17,19,21,22,26,28	No	New	Standalone
Kim et al (2006) [47]	Transtheoretical model	5,6,7,17,19,20,21	Yes	Evolved	Embedded in care
Lorig et al (2006) [45]	Self-efficacy theory,	1,4,7,8,19,21,22,27,28,29,33,34,36	No	Evolved	Standalone
Glasgow et al (2003), Barrera et al (2002), McKay et al (2002) [42-44]	Self-efficacy theory, social support theory	1,2,5,8,10,16,17,19,27-29	Yes	New	Standalone

^a^BCT: Behavioral change technique; see Multimedia Appendix 3.

### Behavioral Change Techniques Used

Only 3 studies explicitly mentioned the BCTs applied [36,37,39]. For the other studies, information on BCTs was extracted from the program description. The frequency of used BCT’s found in the articles is shown in Table 5. The most commonly applied BCT’s were: provide feedback on performance, provide information on consequences of behavior in general, barrier identification/problem solving, provide information on consequences of behavior to the individual, and prompt self-monitoring of behavior. Some of the unused BCTs were shaping, prompting focus on past success, agree behavioral contract, and fear arousal.

### Behavioral Change Techniques Linked to Improved Outcomes

Seven of 13 RCTs reported statistically significant improvements in health behaviors (diet, physical activity/exercise, medication use, smoking) [37,42,44-48]. Nine studies reported statistically significant improvements in clinical outcomes measures, such as glycated hemoglobin (HbA1c), fasting blood glucose, cholesterol, and triglycerides [37,39-43,45-47]. Nine studies reported statistically significant improvements in psychological outcomes, such as depression, diabetes distress, psychosocial well-being, self-efficacy, stress, and communication [35,37-39,42-45,48]. Table 6 provides an overview of the frequency of applied BCTs found to be associated with the statistically significant improvement of study outcomes.

The BCTs provide feedback on performance, provide information on consequences of behavior in general, barrier identification/problem solving, prompt self-monitoring of behavioral outcome, provide information on consequences of behavior to the individual, prompt self-monitoring of behavior, and plan social support/social change were all linked with improvements in health behaviors, clinical outcome measures, and psychological outcomes. Additionally, goal setting (behavior) was linked to improvements in clinical outcomes and facilitate social comparison was associated with improvements in psychological outcomes.

**Table 5:** Frequencies of behavioral change techniques (BCTs) used in the interventions discussed in the articles (n=8).

#	BCT	n	%
1	Provide feedback on performance	8	100
2	Provide information on consequences of behavior in general	7	88
3	Barrier identification/problem solving	7	88
4	Provide information on consequences of behavior to the individual	6	75
5	Prompt self-monitoring of behavior	6	75
6	Prompt self-monitoring of behavioral outcome	6	75
7	Provide instruction on how to perform the behavior	5	63
8	Facilitate social comparison	5	63
9	Plan social support/social change	5	63
10	Goal setting (behavior)	4	50
11	Action planning	4	50
12	Prompt review of behavioral goals	4	50
13	Stress management/emotional control training	4	50
14	Provide normative information about others’ behavior	3	38
15	Model/Demonstrate the behavior	3	38
16	Prompt practice	3	38
17	Use of follow-up prompts	3	38
18	Goal setting (outcome)	2	25
19	Provide rewards contingent on successful behavior	2	25
20	Relapse prevention/coping planning	2	25
21	Provide information about others’ approval	1	13
22	Set graded tasks	1	13
23	Prompt review of outcome goals	1	13
24	Prompt rewards contingent on effort or progress toward behavior	1	13
25	Prompting generalization of a target behavior	1	13
26	Provide information on where and when to perform the behavior	1	13
27	Teach to use prompts/cues	1	13
28	Environmental restructuring	1	13
29	Prompt identification as role model/position advocate	1	13
30	Prompt self-talk	1	13
31	Prompt use of imagery	1	13
32	General communication skills training	1	13
33	Stimulate anticipation of future rewards	1	13
34	Shaping	0	0
35	Prompting focus on past success	0	0
36	Agree behavioral contract	0	0
37	Prompt anticipated regret	0	0
38	Fear arousal	0	0
39	Motivational interviewing	0	0
40	Time management	0	0

**Table 6:** Frequency of behavioral change techniques (BCTs) per improved study outcome.

BCT	Improved health behavior outcomes (n=7)		Improved clinical outcome measures (n=9)		Improved psychological outcomes (n=9)		Combined average percentage
	n	%	n	%	n	%	%
Provide feedback on performance	7	100	9	100	9	100	100
Provide information on consequences of behavior in general	6	86	8	89	9	100	92
Barrier identification/problem solving	6	86	8	89	9	100	92
Prompt self-monitoring of behavioral outcome	6	86	8	89	7	78	84
Provide information on consequences of behavior to the individual	5	71	7	78	8	89	79
Prompt self-monitoring of behavior	5	71	7	78	8	89	79
Plan social support/social change	5	71	6	67	7	78	72
Goal setting (behavior)	5	71	7	78	5	56	68
Prompt review of behavioral goals	4	57	7	78	6	67	67
Facilitate social comparison	4	57	6	67	7	78	67
Action planning	4	57	5	56	3	33	49
Use of follow-up prompts	3	43	3	33	5	56	44
Provide instruction on how to perform the behavior	3	43	4	44	4	44	44
Provide normative information about others’ behavior	3	43	3	33	3	33	36
Stress management/emotional control training	1	14	4	44	4	44	34
Provide rewards contingent on successful behavior	2	29	2	22	2	22	24
Model/Demonstrate the behavior	2	29	1	11	3	33	24
Relapse prevention/coping planning	2	29	2	22	2	22	24
Prompt practice	1	14	2	22	3	33	23
Set graded tasks	2	29	2	22	1	11	21
Goal setting (outcome)	1	14	3	33	1	11	19
Prompt self-talk	1	14	1	11	1	11	12
Prompt use of imagery	1	14.	1	11	1	11	12
Prompt review of outcome goals	0	0	2	22	1	11	11
Teach to use prompts/cues	0	0	2	22	1	11	11
Prompt identification as role model/position advocate	0	0	2	22	1	11	11
Provide information on where and when to perform the behavior	1	14	1	11	0	0	8
Provide information about others’ approval	0	0	1	11	1	11	7
Prompt rewards contingent on effort or progress toward behavior	0	0	0	0	1	11	3
Prompting generalization of a target behavior	0	0	0	0	1	11	3
Environmental restructuring	0	0	0	0	1	11	3
General communication skills training	0	0	0	0	1	11	3
Stimulate anticipation of future rewards	0	0	0	0	1	11	3
Shaping	0	0	0	0	0	0	0
Prompting focus on past success	0	0	0	0	0	0	0
Agree behavioral contract	0	0	0	0	0	0	0
Prompt anticipated regret	0	0	0	0	0	0	0
Fear arousal	0	0	0	0	0	0	0
Motivational interviewing	0	0	0	0	0	0	0
Time management	0	0	0	0	0	0	0

## Discussion

### Overall Findings

To the best of our knowledge, this is the first review of BCT use in online diabetes self-management support programs. This information should prove helpful in designing effective online self-management programs for people with T2DM. We identified 13 RCT studies reporting on 8 different online self-management interventions of which 4 pre-existed as group-based programs. Despite the introduction of the Internet in 1994, only a relatively small number of Internet-based self-management interventions for T2DM patients have been studied. We did find 143 studies on various forms of diabetes telehealth and telecare interventions. These studies were excluded from our review because they did not qualify for our definition of self-management programs. Rather these programs stimulated patients to self-monitor their blood glucose, followed by professional feedback and advice. To our knowledge, these programs do not explicitly prompt or support patients to make decisions [32-34,49].

The majority of the included studies that reported on self-management interventions only gave a very basic description of the program and its background. Indeed, it has been noted before that very few studies provide a detailed description of the actual behavioral change intervention [50-52]. This could be caused by the limited space authors have to describe the intervention in certain journals, making it difficult to replicate the study or allocate an effect size to specific parts of the intervention.

### The Use of Theories and Behavioral Change Techniques

We observed that 7 of 8 interventions were grounded in a theoretical model, of which one of the used models (TTM), although popular, had limited evidence to support its assumptions [53,54]. Self-regulation theory with monitoring, action planning, and evaluating as its key features [55], and social learning theory, characterized by learning in social context [56], were most commonly used to inform development of the online interventions. However, only 3 studies specifically substantiated their choice for the use of specific BCTs to support their intervention [35,37,39]. After distilling the BCTs from the articles, the BCTs feedback on performance, providing information on consequences of behavior, barrier identification/problem solving, and self-monitoring of behavior and outcomes seemed to contribute the most to the effectiveness of the online self-management programs. These techniques also seemed to be key components for healthy eating and increasing physical activity [57], and were also commonly found in offline T2DM self-management programs [14,58]. However, being used frequently is in itself not a guarantee that these BCTs will actually contribute to the improvement of patients’ self-management in a particular domain. Nor does it mean that these BCTs fit the theory that was chosen to guide the intervention [59]. To further the development of effective online self-management interventions for T2DM and other chronic conditions, it is important to understand the underlying learning process [59]. Appropriate use of theories and supporting BCTs can prevent future interventions to be wrongly interpreted or executed by participants thereby improving treatment fidelity. This is particularly important for online programs, where confusion and misinterpretation on the part of the participant is more difficult to detect and address than in a group setting, for example, because of the more distant and static nature of the Web-based intervention. Therefore, it is crucial that the theoretical framework and BCTs are carefully chosen before a Web-based self-management intervention is created [60].

A number of potentially effective BCTs appear to be used rarely or never in online self-management programs for T2DM despite a good theoretical basis. For instance, only a selection of BCTs derived from social theories, which have a great influence on the self-management of T2DM [61], were represented in the reviewed studies that claimed to use these social theories. Although planning social support and having some form of coaching to provide feedback are frequently used, other BCTs that seem to affiliate with social cognitive theories, such as identification of a role model, model/demonstrate the behavior, and provide information about others’ approval, were not frequently used. The same is true for BCTs such as coping planning and use of imagery that have been shown to be effective in stimulating self-management of T2DM in an offline program [62]. Similarly BCTs that seem to be based in the classical and operant conditioning theories (characterized by associations and rewards), such as prompt rewards contingent on effort or progress toward behavior and teach to use prompts/cues, were also barely used, but have shown to be associated with improving physical activity [63]. Just because these theories and BCTs were not used in the reviewed interventions does not mean that they are of no value to an online self-management program.

The question then arises why researchers only use a limited number of BCTs and why the chosen BCTs do not always match the theories underlying their intervention. One explanation could be that current online interventions are being copied from published successful online or offline interventions based on a selection of theories and BCTs. By copying existing self-management programs, other relevant theories and BCTs are slowly phased out, narrowing the spectrum of BCTs used. Another obvious reason why certain BCTs are not being used could be that they are too complex or too technologically demanding and, therefore, too costly to integrate into an online environment. For instance, integrating elements of social support into the intervention, such as a forum, email messaging, and chatting functionality, demands large databases and continuous moderator involvement. The maintenance costs of these parts could influence the choice of using these elements. Finally, another reason for underuse of effective BCTs may be that the development of Web-based interventions for T2DM patients are driven primarily by technological advancements rather than being based on a BCT [64].

### Limitations

The number of articles uncovered in this review was limited to 13 RCTs, covering 8 different diabetes self-management interventions. By only including English studies there is a possibility we limited the amount of available interventions for this review. This, in combination with multiple BCTs used and a variety of outcome measures, made it difficult to allocate an effect size to a specific BCT. Furthermore, because of the fact that self-management interventions contain multiple modules with interactive components, it is difficult to attribute an improvement in a particular study outcome to one specific BCT.

### Conclusions/Future Recommendations

The development of online self-management interventions for T2DM patients brings with it a responsibility of correctly constructing and choosing the working components to specifically target diabetes self-management goals and outcomes. To avoid a further narrowing of applied BCTs, we recommend developers of online self-management programs to not only copy existing successful programs, but also critically review and consider less frequently used BCTs in the context of their theoretical background and the chosen target behaviors.

Ideally, the creation process should follow the order of choosing a theory first, then matching BCTs, and lastly the technology to support the intervention. BCTs can be selected from the taxonomy of 40 BCTs as proposed by Michie et al [27]. By using this strategy, online theory-based self-management programs for T2DM patients can be developed without making unnecessary compromises or biased choices caused by existing technology. Furthermore, reporting detailed information on used theories and BCTs in research protocols and articles will benefit researchers in the creation and understanding of new effective Web-based self-management interventions for T2DM and other chronic disorders.

## Multimedia Appendix 1

Search terms.

## Multimedia Appendix 2

Study quality assessment.

## Multimedia Appendix 3

Behavioral change techniques proposed by Michie et al (2011).

## Abbreviations

AADE: Association of American Diabetes Educators
BCT: behavioral change technique
CG: control group
IG: intervention group
RCT: randomized controlled trial
T1DM: type 1 diabetes mellitus
T2DM: type 2 diabetes mellitus
TTM: transtheoretical model
