# Supplementary material for: Use of Behavioral Change Techniques in Web-Based Self-Management Programs for Type 2 Diabetes Patients: Systematic Review
Source: J Med Internet Res. 2013 Dec 13;15(12):e279. doi: 10.2196/jmir.2800 (PMC3869055; doi:10.2196/jmir.2800)
Supplement: Supplementary file 1 [file jmir_v15i12e279_app1.pdf]

Appendix 1: Search terms

PubMed:

#1 Diabetes
"Glucose Metabolism Disorders"[Mesh:NoExp] OR "Diabetes Mellitus"[Mesh:NoExp] OR "Diabetes Mellitus, Type 1"[Mesh] OR "Diabetes Mellitus, Type 2"[Mesh] OR "Diabetes Complications"[Mesh] OR "Hyperglycemia"[Mesh] OR "Hypoglycemia"[Mesh] OR diabet*[tiab] OR "dm 1"[tiab] OR DM1[tiab] OR DMI[tiab] OR T1DM[tiab] OR TIDM[tiab] OR iddm[tiab] OR "insulin dependent"[tiab] OR "dm 2"[tiab] OR DM2[tiab] OR DMII[tiab] OR T2DM[tiab] OR TIIDM[tiab] OR NIDDM[tiab] OR "Non Insulin Dependent"[tiab] OR "LADA"[tiab] OR MODY[tiab] OR hyperglycemi*[tiab] OR hypoglycemi*[tiab] OR "glucose intolerance"[tiab]
#2 E-health
"Internet"[Mesh] OR "e-health"[tiab] OR Ehealth[tiab] OR Online[tiab] OR "web-based"[tiab] OR telemedicine[tiab] OR computer*[tiab] OR "mobile phone"[tiab] OR GSM[tiab] OR "cell phone"[tiab] OR website[tiab] OR internet[tiab] OR teleconsultation[tiab] OR electronic[tiab] OR "ICT"[tiab] OR phone*[tiab] OR iphone*[tiab] OR ipad*[tiab] OR android*[tiab] OR game[tiab] OR gaming[tiab] OR virtual[tiab] OR "mhealth"[tiab] OR "m-health"[tiab]

EMBASE

#1 Diabetes type
'disorders of carbohydrate metabolism'/de OR 'diabetes mellitus'/de OR 'insulin dependent diabetes mellitus'/exp OR 'Wolfram syndrome'/exp OR 'non insulin dependent diabetes mellitus'/exp OR 'lipoatrophic diabetes mellitus'/exp OR 'diabetic angiopathy'/exp OR 'diabetic cardiomyopathy'/exp OR 'diabetic coma'/exp OR 'diabetic foot'/exp OR 'diabetic ketoacidosis'/exp OR 'diabetic hypertension'/exp OR 'diabetic macular edema'/exp/mj OR 'diabetic nephropathy'/exp/mj OR 'diabetic neuropathy'/exp/mj OR 'diabetic obesity'/exp/mj OR 'diabetic retinopathy'/exp/mj OR 'impaired glucose tolerance'/exp/mj OR 'maturity onset diabetes mellitus'/exp/mj OR 'hyperglycemia'/exp/mj OR 'diabetic patient'/exp
#2 E-health
'Internet'/exp OR 'electronic data interchange'/exp OR 'online analysis'/exp OR 'online monitoring'/exp OR 'mass communication'/exp OR 'teleconsultation'/exp

Cochrane Library

#1 Diabetes
Glucose Metabolism Disorders OR Diabet* OR "Hyperglycemia" OR "dm 1" OR DM1 OR DMI OR T1DM OR TIDM OR iddm OR "insulin dependent" OR "dm 2" OR DM2 OR DMII OR T2DM OR TIIDM OR NIDDM OR "Non Insulin Dependent" OR MODY OR "glucose intolerance"
#2 E-health

Internet OR Communication OR Medical Informatics Applications OR Internet OR e-health OR Ehealth OR Online OR "web-based" OR telemedicine OR computer* OR "mobile phone" OR GSM OR "cell phone" OR website OR internet OR teleconsultation OR electronic OR "ICT" OR phone* OR iphone* OR ipad* OR android* OR game OR gaming OR virtual OR mhealth OR "m-health"

PsycINFO:

#1 Diabetes
DE "Diabetes" OR MM "Diabetes Mellitus" OR DE "Diabetes Mellitus" OR MM "Diabetes" OR DE "Hyperglycemia" OR DE "Hypoglycemia" OR (diabet* OR (type AND (1 OR I) AND diabetes) OR "diabetes 1" OR "diabetes I" OR "dm 1" OR DM1 OR DMI OR T1DM OR TIDM OR iddm OR "insulin dependent" OR (type AND (2 OR II) AND diabetes) OR "diabetes 2" OR "diabetes II" OR "dm 2" OR DM2 OR DMII OR T2DM OR TIIDM OR NIDDM OR "Non Insulin Dependent" OR MODY OR hyperglycemia OR "glucose intolerance")
#2 E-health
DE "Electronic Communication" OR DE "Computer Mediated Communication" OR DE "Internet Addiction" OR DE "Online Therapy" OR DE "Telecommunications Media" OR DE "Radio" OR DE "Telephone Systems" OR DE "Television" OR DE "Television Advertising" OR DE "Telemedicine" OR DE "Websites" OR DE "Online Therapy" OR DE "Treatment" OR DE "Computer Applications" OR DE "Computer Assisted Therapy" OR DE "Computer Mediated Communication" OR DE "Health Care Seeking Behavior" OR DE "Internet" OR DE "Psychotherapy" OR DE "Telecommunications Media" OR DE "Cellular Phones" OR Internet OR "e-health" OR Ehealth OR Online OR "web-based" OR telemedicine OR computer* OR "mobile phone" OR GSM OR "cell phone" OR website OR teleconsultation OR "ICT" OR phone* OR iphone* OR ipad* OR android* OR game OR gaming OR virtual OR "mhealth" OR "m-health"

Cinahl Library

#1 Diabetes
(MH "Diabetes Education/ED/ES/PF/TD") OR (MH "Diabetes Educators") OR (MH "Diabetes Mellitus/PF/TH/TD/RF/ED") OR (MH "Diabetes Mellitus, Gestational") OR (MH "Diabetes Mellitus, Type 1") OR (MH "Diabetes Mellitus, Type 2") OR (MH "Diabetes UK") OR (MH "Diabetic Diet") OR (MH "Diabetic Patients") OR (MH "Hypoglycemia") OR (MH "Hypoglycemia Management (Iowa NIC)") OR (MH "Hyperglycemia") OR (MH "Hyperglycemia Management (Iowa NIC)") OR (diabet* OR (type AND (1 OR I) AND diabetes) OR "diabetes 1" OR "diabetes I" OR "dm 1" OR DM1 OR DMI OR T1DM OR TIDM OR iddm OR "insulin dependent" OR (type AND (2 OR II) AND diabetes) OR "diabetes 2" OR "diabetes II" OR "dm 2" OR DM2 OR DMII OR T2DM OR TIIDM OR NIDDM OR "Non Insulin Dependent" OR MODY OR hyperglycemia OR "glucose intolerance")
#2 E-health
(MH "Internet") OR (MH "Telephone") OR (MH "Telehealth+") OR (MH "Telecommunications+") OR (MH "Mail") OR (MH "Electronic Mail") OR (MH "Online Services") OR (MH "Telehealth+") OR (MH "Telemedicine+") OR (MH "Remote

Consultation") OR (MH "Electronic Bulletin Boards") OR (MH "Instant Messaging") OR (MH "Interactive Voice Response Systems") OR (MH "Telecommuting") OR (MH "Wireless Communications") OR (MH "Videoconferencing") OR (MH "Telephone") OR (MH "Teleconferencing") OR Internet OR "e-health" OR Ehealth OR Online OR "web-based" OR telemedicine OR computer* OR "mobile phone" OR GSM OR "cell phone" OR website OR internet OR teleconsultation OR "ICT" OR phone* OR iphone* OR ipad* OR android* OR game OR gaming OR virtual OR "mhealth" OR "m-health"
