# Supplementary material for: Use of Behavioral Change Techniques in Web-Based Self-Management Programs for Type 2 Diabetes Patients: Systematic Review
Source: J Med Internet Res. 2013 Dec 13;15(12):e279. doi: 10.2196/jmir.2800 (PMC3869055; doi:10.2196/jmir.2800)
Supplement: Supplementary file 2 [file jmir_v15i12e279_app2.pdf]

Appendix 2: Study quality assessment

		Glasgow (2012) [37]	van Bastelaar (2011) [35]	Bond (2010) [38]	Lorig (2010) [39]	Glasgow (2010) [46]	Wangberg (2008) [48]	Bond (2007) [40]	Bond (2006) [41]	Kim (2006) [47]	Lorig (2006) [45]	Glasgow (2003) [42]	Barrera (2002) [43]	McKay (2002) [44]
a	Eligibility criteria specified	yes	yes	yes	yes	yes	yes	yes	yes	yes	yes	yes	yes	yes
b1	Randomization performed?	yes	yes	yes	yes	yes	yes	yes	yes	yes	yes	yes	yes	yes
c	Groups similar at baseline?	yes	yes	yes	yes	yes	yes	yes	unclear	yes	yes	yes	yes	yes
d	Interventions explicitly described	yes	yes	yes	yes	yes	yes	yes	yes	yes	yes	yes	yes	yes
e	Care provider blinded to the intervention?	no	no	unclear	uncear	no	unclear	unclear	unclear	no	unclear	unclear	unclear	unclear
f	Were co-interventions avoided or comparable?	yes	yes	yes	yes	yes	yes	yes	unclear	yes	yes	yes	yes	yes
g	Compliance acceptable in all groups?	yes	yes	yes	yes	yes	no	yes	yes	yes	yes	yes	yes	yes
h	Patient blinded to the intervention?	unclear	no	unclear	uncear	unclear	unclear	unclear	unclear	no	unclear	unclear	unclear	unclear
i	Researcher blinded to the intervention?	unclear	unclear	unclear	uncear	unclear	unclear	yes	unclear	no	unclear	unclear	unclear	unclear
j	Outcome measures relevant?	yes	yes	yes	yes	yes	yes	yes	yes	yes	yes	yes	yes	yes
k	Were adverse effects described?	yes	yes	yes	yes	yes	yes	yes	no	yes	yes	yes	yes	yes
l	Withdrawal/dropout rate described and acceptable?	yes	yes	yes	yes	yes	yes	yes	yes	yes	yes	yes	yes	yes
m1	Short-term follow-up measurement?	yes	yes	yes	yes	yes	yes	yes	yes	yes	yes	no	yes	yes
m2	Long-term follow-up measurement?	yes	yes	no	yes	no	no	no	no	no	yes	yes	no	no
n	Timing of outcome assessment in both groups comparable?	yes	yes	yes	yes	yes	yes	yes	yes	yes	yes	yes	yes	yes
o	Sample size for each group described?	yes	yes	yes	yes	yes	yes	yes	yes	yes	yes	no	yes	yes
p	Intention-to-treat analysis?	yes	yes	no	no	yes	no	no	no	no	no	no	no	no
q	Were point estimates and measures of variability presented for the primary outcome measures?	yes	yes	yes	yes	yes	yes	yes	no	yes	yes	yes	yes	yes
	Number of criteria met (maximum of 18 criteria)	83%	83%	72%	78%	78%	67%	78%	50%	72%	78%	67%	72%	72%
