# Supplementary material for: Use of Behavioral Change Techniques in Web-Based Self-Management Programs for Type 2 Diabetes Patients: Systematic Review
Source: J Med Internet Res. 2013 Dec 13;15(12):e279. doi: 10.2196/jmir.2800 (PMC3869055; doi:10.2196/jmir.2800)
Supplement: Supplementary file 3 [file jmir_v15i12e279_app3.pdf]

Appendix 3: Behavioral change techniques proposed by Michie et al. (2011)

1. Provide information on consequences of behaviour in general
2. Provide information on consequences of behaviour to the individual
3. Provide information about others’ approval
4. Provide normative information about others’ behaviour
5. Goal setting (behaviour)
6. Goal setting (outcome)
7. Action planning
8. Barrier identification/problem solving
9. Set graded tasks
10. Prompt review of behavioural goals
11. Prompt review of outcome goals
12. Prompt rewards contingent on effort or progress towards behaviour
13. Provide rewards contingent on successful behaviour
14. Shaping
15. Prompting generalisation of a target behaviour
16. Prompt self-monitoring of behaviour
17. Prompt self-monitoring of behavioural outcome
18. Prompting focus on past success
19. Provide feedback on performance
20. Provide information on where and when to perform the behaviour
21. Provide instruction on how to perform the behaviour
22. Model/Demonstrate the behaviour
23. Teach to use prompts/cues
24. Environmental restructuring
25. Agree behavioural contract
26. Prompt practice
27. Use of follow-up prompts
28. Facilitate social comparison
29. Plan social support/social change
30. Prompt identification as role model/position advocate
31. Prompt anticipated regret
32. Fear arousal
33. Prompt self talk
34. Prompt use of imagery
35. Relapse prevention/coping planning
36. Stress management/emotional control training
37. Motivational interviewing
38. Time management
39. General communication skills training
40. Stimulate anticipation of future rewards
